# The Procoagulant Activity of Emoxilane^®^: A New Appealing Therapeutic Use in Epistaxis of the Combination of Sodium Hyaluronate, Silver Salt, α-tocopherol and D-panthenol

**DOI:** 10.3390/life11090992

**Published:** 2021-09-21

**Authors:** Raffaella Belvedere, Nunzia Novizio, Daniela Eletto, Amalia Porta, Antonino Bagnulo, Andrea Cerciello, Umberto Di Maio, Antonello Petrella

**Affiliations:** 1 Department of Pharmacy, University of Salerno, Via Giovanni Paolo II 132, 84084 Fisciano, Italy; rbelvedere@unisa.it (R.B.); nnovizio@unisa.it (N.N.); daeletto@unisa.it (D.E.); aporta@unisa.it (A.P.); 2 Neilos Srl, Via Bagnulo 95, 80063 Piano di Sorrento, Italy; a.bagnulo@neilos2015.com (A.B.); a.cerciello@neilos2015.com (A.C.); 3 Shedir Pharma Group Spa, Via Bagnulo 95, 80063 Piano di Sorrento, Italy; u.dimaio@shedirpharmagroup.com

**Keywords:** Emoxilane^®^, epistaxis, sodium hyaluronate, silver nitrate, α-tocopherol, D-panthenol, oxidation, antimicrobial effects, plasmin activity, thrombin levels

## Abstract

Epistaxis is one of the most frequent hemorrhages resulting from local or systemic factors. Its management without hospitalization has prompted an interest in locally applied hemostatic agents. Generally, the therapy approaches involve sprays or creams acting as a physical barrier, even used as tampons or gauze. In this study, we have investigated the activity of Emoxilane^®^, a combination of sodium hyaluronate, silver salt, α-tocopherol acetate and D-panthenol, which is known to be able to separately act in a different biological manner. Our in vitro results, obtained on endothelial and nasal epithelial cells, have shown that the association of these molecules presented a notable antioxidant activity mainly due to the α-tocopherol and D-panthenol and a significant antimicrobial role thanks to the silver compound. Moreover, remarkable hemostatic activity was found by evaluating plasmin inhibition attributable to the sodium hyaluronate. Interestingly, on human plasma, we have confirmed that Emoxilane^®^ strongly induced the increase of thrombin levels. These data suggest that the use of this association could represent an appealing pharmacological approach to actively induce hemostasis during epistaxis. Our future perspective will aim to the creation of a formulation for an easy topical application in the nose which is able to contrast the bleeding.

## 1. Introduction

Hemostasis is a process aimed to arrest a hemorrhage after damage to a blood vessel; when this damage occurs in the nasal mucosa, generating nose bleeding, it is defined as epistaxis. This latter can result from local or systemic factors, mainly traumatic, neoplastic, hematological, inflammatory ones, or therapies with anticoagulants and antiplatelet drugs [1]. No uniform guidelines exist for diagnostic and therapeutic procedures in patients with epistaxis. However, the management without the need for hospital admission has prompted growing interest [2]. Generally, the treatment involves primary care mainly based on compression through a nasal tampon or gauze. Next, anterior rhinoscopy and/or endoscopy are eventually required. As a final step, refractory bleeding makes endoscopic surgery necessary as cauterization or ligation of the bleeding blood vessel performed by an otolaryngologist. If even these techniques are not successful, recourse must be made to the intervention of radiologists for digital subtraction angiography (DSA) and embolization of the blood vessel that causes the bleeding [3,4].

Commonly, topical antimicrobial agents, based for the most part on the activity of silver compounds, should also be used since the prevention of contaminations must always be observed [5]. Therefore, all these kinds of approaches are based on the establishment of barrier effects, thus they passively oppose to bleeding. In the last few years, several efforts were made to achieve active biological processes which could intervene and favor endothelial repair. In this regard, one of the preferential advances has involved the use of vitamins and minerals as tocopherol, ascorbic acid, selenium, carotenoids, and phenolic/polyphenolic compounds. Indeed, they are considered some of the major contributors for the maintenance of a favorable redox environment preventing vascular endothelial dysfunction from the scavenger reactive oxygen species [6,7]. Moreover, the integrity of endothelial and epithelial components can be also sustained by the protective effect of several kinds of formulations based on DL-panthenol (pro vitamin B5) and on hyaloronic acid. Mostly, this glycosaminoglycan has appeared as one on the main component of topical nasal preparations because its well-known functions during the regeneration process [8,9,10,11]. 

Thus, the management of epistaxis requires local treatments or medication with the primary aim of the vasoconstriction and/or pro-coagulant action. Then it is necessary to activate the epithelial cells surrounding the injured vessel to obtain a correct repair of the whole area. Furthermore, a moist environment may assist the restoring process. Finally, an anti-bacterial action is preferable since there is an increased rate of nasal bacterial infections in epistaxis patients. For all these reasons, we chose to investigate on the association named Emoxilane^®^ which represents a new and complete combination of sodium hyarulonate, silver salt, α-tocopherol acetate, and D-panthenol that we have thought to join together as it is potentially able to act in a different manner on each of the highlighted needs. In this study, each molecular component was analyzed as a single one and in several kinds of combination, until the definitive assessment of Emoxilane^®^. As a preliminary investigation, we evaluated in vitro the antioxidant and antimicrobial roles. We also found the attractive effects on the inhibition of fibrinolysis, by assessing the plasmin in vitro activity, and the increase of thrombin levels in human plasma. Thus, this study aimed to create solid foundations for the future development of Emoxilane^®^ as a nasal topical formulation with multiple beneficial activities contrasting nose bleeding.

## 2. Materials and Methods

### 2.1. Cell Cultures

Human umbilical vein endothelial cells (HUVEC) (ATCC^®^ PCS-100-010™, Manassas, VA, USA) were cultured as reported in [12] until passage 10. Human nasal epithelial cells (HNEpC) were purchased from PromoCell (Atlanta, GA, USA) and cultured in the ready-to-use airway epithelial cell growth medium (PromoCell; Atlanta, GA, USA). Cells were stained at 37 °C in 5–95% CO_2_ air humidified atmosphere.

### 2.2. Preparation of Sample Solutions

All the substances used in this work were provided from Shedir Pharma srl. Sodium hyaluronate (SA) and silver nitrate (Ag), as powders, were dissolved in sterile deionized water to obtain 2 mg/mL and 100 mg/mL stock solutions, respectively. D-panthenol (Pant) was provided as gel and dissolved in sterile bi-distilled deionized water starting from a 10% *w*/*v* solution. Finally, the powder of α-Tocopherol acetate (Toc) was solubilized in sterile bi-distilled deionized water with 10% ethanol as 1M stock solution. Then, these solutions were diluted in cell growth medium or PBS 1× reaching the needed final concentrations as follows:

- SA: 100 µg/mL; 200 µg/mL;
- Ag: 15 µg/mL; 30 µg/mL;
- Toc: 20 µM; 40 µM;
- Pant: 1% *w*/*v*: 2% *w*/*v*.

Due to the lack of differences between the two tested concentrations for each substance taken alone, only one concentration was shown (SA: 200 µg/mL; Ag: 30 µg/mL; Toc: 40 µM; Pant: 1% *w*/*v*) both as a single treatment and for the mixtures. 

### 2.3. 3-(4,5-dimethylthiazol-2-yl)-2,5-diphenyltetrazolium Bromide (MTT) Assay

After the treatments, HUVEC and HNEpC cells were harvested at the indicated times (24, 48, and 72 h) and cell viability was calculated as previously described [13]. The optical density (OD) of each well was measured with a spectrophotometer (Titertek Multiskan MCC/340) equipped with a 620 nm filter.

### 2.4. 1,1-diphenyl-2-picrylhydrazyl (DPPH) Assay

Antioxidant activity was determined through spectrophotometry using DPPH scavenging radical assay. The 0.04 mg/mL DPPH (Sigma-Aldrich Co., St. Louis, MO, USA) solution was prepared in methanol; this dilution was prepared in order to reach the concentration that gives an absorbance of about 2.5 at 520 nm when measured at the multi-well-reading spectrophotometer (Titertek Multiskan MCC/340). In a 96 multi-well, 150 µL of each tested substance, as a single one and in combination, were mixed with 150 µL of DPPH solution. After incubation for 30 min in the dark at room temperature, absorbance at 520 nm was used to calculate radical scavenging activity (% of inhibition) as described in [14]. 

### 2.5. 2′,7′-Dichlorodihydrofluorescein Diacetate (DCHF-DA) Assay

Intracellular oxidative stress was detected using DCFH-DA (Sigma-Aldrich Co., St. Louis, MO, USA). HUVEC and HNEpC cells were plated at 1.5 × 105/well and treated for 24 h with the tested conditions after a further 24 h pretreatment with CoCl_2_ 100 µM used as oxidant stimulus. Then, a subsequent incubation with DCHF-DA 10 μM for 15 min at 37 °C in the dark was performed. Cells were harvested and evaluated through the Becton Dickinson FACScan flow cytometer (BD FacsCalibur, Milan, Italy) using the Cells Quest program [15].

### 2.6. Antimicrobial Activity

The inhibition of microbial growth of each solution was evaluated against *Staphylococcus aureus* (ATCC 6538), *Staphylococcus epidermidis* (ATCC 12228), *Pseudomonas aeruginosa* (ATCC 27853), and *Escherichia coli* (ATCC 8739). To determine the in vitro inhibitory effect of each molecule, the indicated bacterial strains were incubated with the test solutions. In details, from overnight cultures in Mueller–Hinton agar (Becton Dickinson and Company, Franklin Lakes, NJ, USA) bacteria were diluted at a concentration of ≈5 × 105 CFU/mL in Mueller–Hinton broth containing the respective solutions listed in Section 2.2. Bacterial cultures were incubated in air for 24 h at 37 °C and the inhibitory effect of each condition was then determined by reading each bacterial culture at 600 nm and comparing their viability to untreated control bacteria.

### 2.7. Plasmin Activity Assay

The plasmin activity was assayed in cell supernatants collected from HUVEC and HNEpC (seeded in a 24-well plastic plate at 1.5 × 10^5^ cells per well) as reported in [16]. Beside the solutions of interest, the mesoglycan was also used at 300 µg/mL for 24 h as positive control and the apotrinin (Sigma-Aldrich; St. Louis, MO, USA), as a serine protease inhibitor, administered at 30 µM, 10 min before reading. The samples were analyzed through the Plasmin Activity Assay kit (Abcam, Cambridge, UK) following the manufacturer’s instructions and adapted to cell media. Particularly, the standard curve was created, starting from a plasmin concentration of 0 ng/well (in 50 µL of standard volume/well) to 75 ng/well. A total of 50 µL of each experimental point was used for reading in triplicate. A total of 50 µL of reaction mix (plasmin assay buffer + plasmin substrate) was added to standard and sample wells. The output fluorescence was evaluated as Ex/Em = 360/450 nm at the EnSight Multimode Plate Reader (PerkinElmer; Waltham, MA, USA) in a kinetic mode (a reading each 2 min from 10 to 20 min of plate incubation at 37 °C). The amount of plasmin was calculated, as previously reported. 

### 2.8. Thrombin Levels Measurement 

Plasma samples were obtained from 6 healthy donors, aged >18 years, collected at San Giovanni di Dio e Ruggi d’Aragona University Hospital, Salerno (Italy). Samples were incubated with the substances for 2 min at room temperature (RT) and, later, thrombin levels were analyzed on EDTA human plasma through the use of the Human Thrombin SimpleStep ELISA kit (Abcam, Cambridge, UK) following the manufacturer’s instructions. As for the plasmin activity assay, the interpolation to standard curve was performed on GraphPad Prism software (version 5.03; GraphPad Software Inc.; San Diego, CA, USA). 

### 2.9. Statistical Analysis

All results are the mean ± SD (standard deviation) of at least 3 experiments performed in triplicate. Statistical comparisons between groups were made using two-tailed t-test comparing two variables. Differences were considered significant if *p* < 0.05, *p* < 0.01 and *p* < 0.001.

## 3. Results

### 3.1. All the Substances of Our Interest, Alone or in Different Kind of Combination, Did Not Result Cytotoxic 

**Figure 1:**
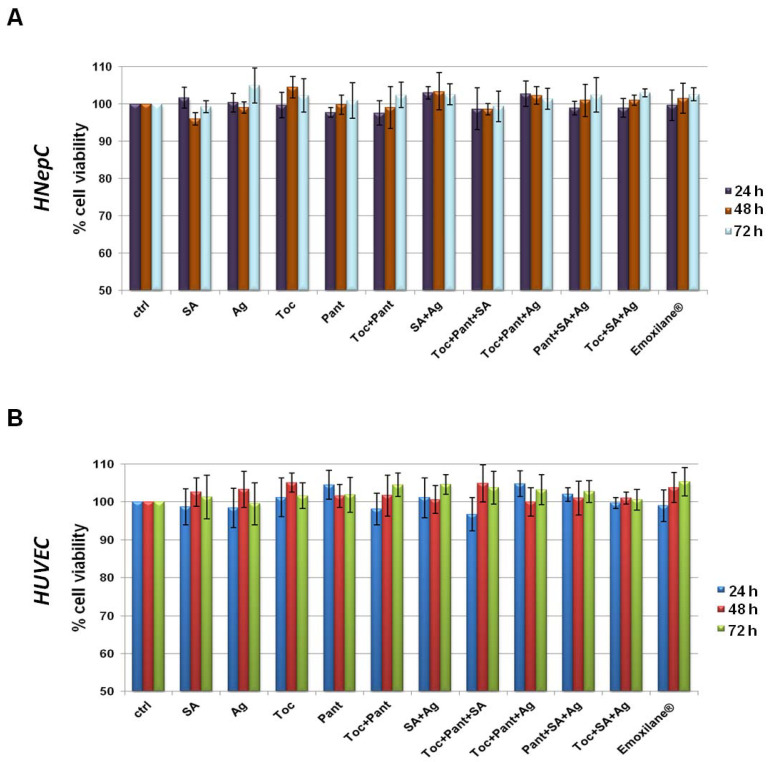
MTT assay was performed after HNEpC (**A**) and HUVEC (**B**) were treated for 24, 48, and 72 h with SA (200 µg/mL), Ag (30 µg/mL), Toc (40 µM), Pant (1% *w*/*v*), Toc+Pant, SA+Ag, Toc+Pant+SA, Toc+Pant+Ag, Pant+SA+Ag, Toc+SA+Ag, Toc+Pan+SA+Ag (Emoxilane^®^). Data are represented as mean ± SD. No statistically significant differences were determined.

In order to assess the biological effects described below concerning the molecules, we have chosen to investigate in this work. First, we evaluated the viability of HNEpC and HUVEC cells. Through the MTT assay, we showed that none of these substances had any kind of cytotoxic effects on both cell lines (Figure 1A,B for HNEpC and HUVEC cells, respectively). Interestingly, all the mixtures in which we combined SA 200 µg/mL, Ag 30 µg/mL, Toc 40 µM and Pant 1% *w*/*v* retained no toxic effects at 24, 48 and 72 h. This data allowed us to continue our research.

### 3.2. The Combination Emoxilane^®^ Showed Marked Antioxidant Effects

**Figure 2:**
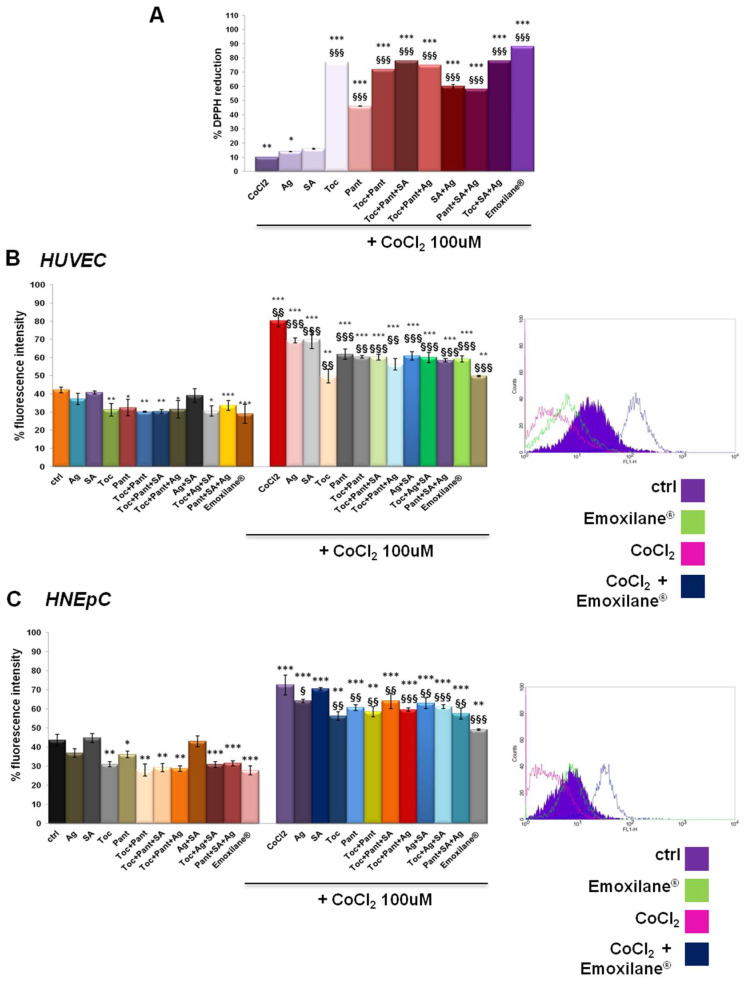
(**A**) DPPH assay performed in presence of CoCl_2_ 100 µM used as oxidant control. DCF-DA assay on HUVEC (**B**) and HNEpC (**C**) treated or not for 24 h with CoCl_2_ 100 µM and 24 h more with SA (200 µg/mL), Ag (30 µg/mL), Toc (40 µM), Pant (1% *w*/*v*), Toc+Pant, SA+Ag, Toc+Pant+SA, Toc+Pant+Ag, Pant+SA+Ag, Toc+SA+Ag, Toc+Pan+SA+Ag (Emoxilane^®^). In both cases, representative flow histogram plots are reported for only cells treated or not with CoCl_2_ and then with Emoxilane^®^ Data are represented as mean ± SD. * *p* < 0.05; ** *p* < 0.01; *** *p* < 0.001 vs. ctrl; § *p* < 0.05; §§ *p* < 0.01; §§§ *p* < 0.001 vs. CoCl_2._

In order to evaluate the antioxidant effect of both single and combined substances, we performed a DPPH assay which is based on the capacity of this organic molecule to change color (from purple to yellow) in the presence of reducing (antioxidants) substances. The assay was carried out in the presence of a strong oxidizing agent such as cobalt chloride (CoCl_2_) 100 µM. The results reported in Figure 2A show the strong antioxidant power of α-tocopherol, D-panthenol and, to a lesser extent, silver salt both alone and in combination. Sodium hyaluronate had no effects. 

Next, the antioxidant effect on HUVEC and HNEpC cells was analyzed by a DCF-DA assay, a fluorescent probe that detects reactive oxygen species (the fluorescence intensity increases in direct association of to the oxidant activity). Cell lines were incubated for 15 min in the dark after the chosen treatments for 24 h either alone or after 24 h of pretreatment with 100 µM CoCl_2_. In Figure 2B, the bar graph represents the results of the fluorescence intensity on HUVEC cells evaluated for all the experimental points. On the other hand, the plot is representative of the fluorescence profile for Emoxilane^®^ with or without CoCl_2_ against the relative controls as shown in the adjacent legend. The positive effect, in terms of contrasting the degree of oxidation in vitro, of the α-tocopherol and D-panthenol, both alone and in association with silver salt and sodium hyaluronate, is confirmed. Moreover, in presence of the oxidant insult, however, the silver compound maintains a marked antioxidant efficacy in case of co-administration with α-tocopherol and D-panthenol. Similarly, the same experiments with DCF-DA conducted on HNepC nasal epithelial cells also showed a significant antioxidant power by silver nitrate, α-tocopherol and D-panthenol (Figure 2C). As example, the flow cytometry analysis of the experimental points shown in the legend are also shown. In the presence of Emoxilane^®^, the intensity of the cell fluorescence decreases significantly compared not only to treatment with CoCl_2_ only but also on untreated cells. In each case, of sodium hyaluronate no antioxidant effect depends.

### 3.3. Emoxilane^®^ Presented a Significant Antimicrobial Activity Thanks to the Presence of Silver Compound

**Figure 3:**
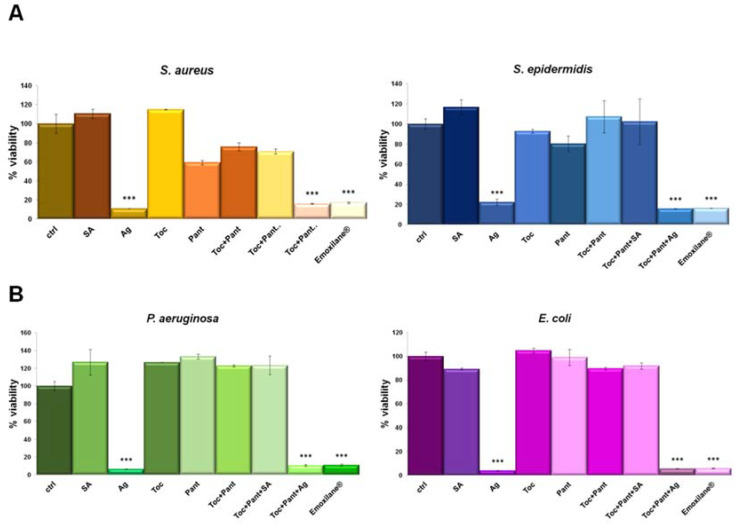
(**A***) S. aureus*, *S. epidermidis*, *E. coli* and *P. aeruginosa* (**B**) were grown for 24 h in presence of the indicated test solutions: SA (200 µg/mL), Ag (30 µg/mL), Toc (40 µM), Pant (1% *w*/*v*), Toc+Pant, Toc+Pant+SA, Toc+Pant+Ag, Toc+Pan+SA+Ag (Emoxilane^®^). Bacterial cultures were measured at 600 nm and the results expressed as mean ± SD. *** *p* < 0.001 vs. ctrl.

Antibacterial activity was evaluated against two gram-positive (*Staphylococcus aureus, Staphylococcus epidermidis*) (Figure 3A) and two gram-negative (*Escherichia coli and Pseudomonas aeruginosa*) (Figure 3B) bacterial strains. Among all the conditions, the most significant inhibitory effect (>80%) was observed with silver salt, either alone or in combination. Additionally, the antimicrobial property of silver was similar against all the tested bacteria and in all the conditions, suggesting that silver retains its efficacy when combined with the indicated compounds.

### 3.4. Emoxilane^®^ Mediates Anti-Fibrinolytic Effects by Inhibiting Plasmin Activity

**Figure 4:**
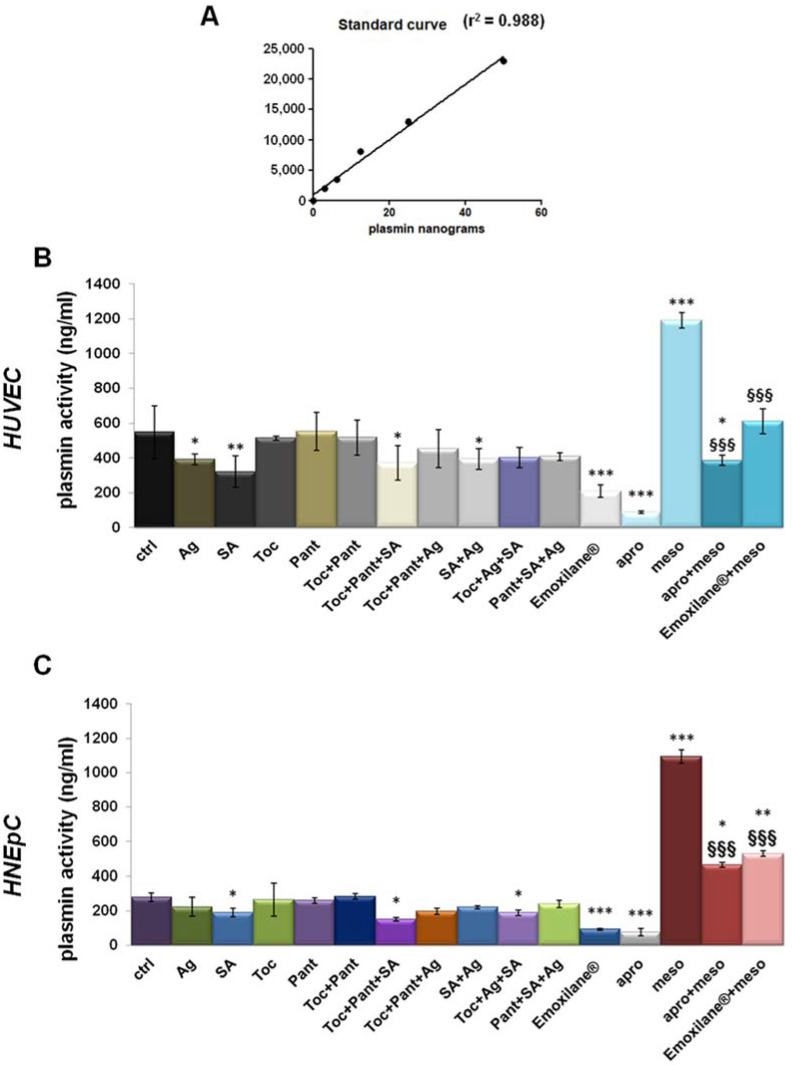
(**A**) Standard curve created from 0 ng to 75 ng in 50 µL of standard volume/well of plasmin concentration (standard enzyme provided by the manufacturer). Histograms representing the ng of plasmin in 50 µL of supernatant samples obtained from HUVEC (**B**) and HNEpC (**C**) cells treated or not with SA (200 µg/mL), Ag (30 µg/mL), Toc (40 µM), Pant (1% *w*/*v*), Toc+Pant, SA+Ag, Toc+Pant+SA, Toc+Pant+Ag, Pant+SA+Ag, Toc+SA+Ag, Toc+Pan+SA+Ag Emoxilane^®^). The association was tested also against mesoglycan 300 µg/mL for 24 h which was evaluated in presence or not of aprotinin 30 µM administered to supernatant samples 10 min before reading. (**C**) Western blotting of total protein extract from HUVEC treated or not with mesoglycan (300 μg/mL; 24 h), siRNAs against ANXA2 (100 nM; 48 h), and siANXA2s and mesoglycan (100 nM; 48 h and 300 μg/mL; 24 h, respectively). The data represent a mean of *n* = 3 independent experiments ± SD, * *p* < 0.05; ** *p* < 001; *** *p* < 0.001 vs. untreated control; §§§ *p* < 0.001 vs. mesoglycan treated cells.

Next, we observed the effects of the tested molecules alone or in combination on the activity of plasmin delivered from plasminogen in cell supernatants as reported in [16]. First, in Figure 4A, we presented the graph of the standard curve. Figure 4B,C showed the results obtained on HUVEC and HNEpC cells, respectively. For both cell lines a slight inhibition of plasmin activity appeared in presence of sodium hyaluronate both alone or in combination with α-tocopherol and D-panthenol. Interestingly, the whole association Emoxilane^®^ drastically blocked the release of the enzyme, in a similar way than the apotrinin, the well-known serine protease we used to corroborate our results. Furthermore, we also proved the anti-fibrinolytic effects of Emoxilane^®^ by using the mesoglycan 300 µg/mL as positive control. Indeed, mesoglycan was previously shown as a strong activator of plasmin in HUVEC cell supernatant [15] against which apotrinin confirmed its inhibitory effect. Notably, also in the case of mesoglycan administration, Emoxilane^®^ demonstrated its efficacy on both HUVEC and HNEpC cells inducing a strong decrease of fluorescence signal.

### 3.5. Thrombin Levels Increased in Human Plasma in Presence of Emoxilane^®^

**Figure 5:**
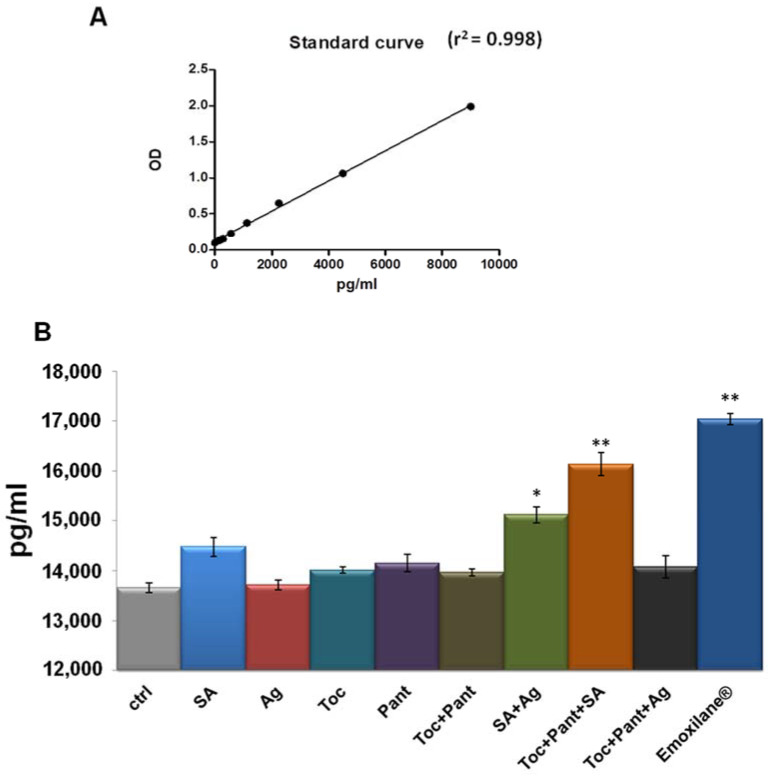
(**A**) Standard curve created from 0 pg to 9000 pg in 100 µL of standard volume/well of thrombin concentration (standard enzyme provided by the manufacturer). (**B**) Histogram representing the pg/mL of thrombin in 100 µL of human plasma samples. Treatments were performed with SA (200 µg/mL), Ag (30 µg/mL), Toc (40 µM), Pant (1% *w*/*v*), Toc+Pant, SA+Ag, Toc+Pant+SA, Toc+Pant+Ag, Toc+Pan+SA+Ag (Emoxilane^®^), with respect to not treated sample, for 2 min before incubation performed as indicated by kit manufacturer. The data represent a mean of *n* = 4 independent experiments ± SD, * *p* < 0.05; ** *p* < 001 vs. untreated control.

Finally, human plasma from healthy donors was used as a sample to evaluate the variation of thrombin levels in the presence of Emoxilane^®^ in the manner reported in the Material and Methods Section. We have started from the finding of the decrease of cell plasmin activity, linked to the inhibition of fibrin clot degradation. Here, this specific kind of analysis allowed us to establish that thrombin, known to be strongly and directly correlated to fibrin clot formation [17], resulted as affected by the molecules of our interest. In particular, the amount of thrombin generated from pro-thrombin was revealed referring to the standard curve reported in Figure 5A. In this way, we have shown that Emoxilane^®^ was notably able to induce an increase of the pg of thrombin on ml of plasma. This ability appeared to be linked to sodium hyaluronate which, in turn, acted more efficiently if associated to the silver salt or with the mixture of α-tocopherol/D-panthenol (Figure 5B).

## 4. Discussion

Epistaxis is a common problem in emergency medicine with a possible serious evolution. Up to 60% of the population is estimated to have had at least one episode of epistaxis in their lives. Of this group, 6% seek medical care to treat epistaxis, with a very low percentage of hospitalization. Most cases of epistaxis occur in children younger than 10 years [18]. The comprehensive management of nosebleeds assumes several recommendations also concerning the knowledge of the personal history of bleeding disorders in patients and the eventual use of anticoagulant or antiplatelet medications, or intranasal drugs. Furthermore, the clinician should treat the epistaxis with topical vasoconstrictors, nasal cautery, and moisturizing or lubricating agents [19]. Nowadays, the medical practices are usually based on the nasal insertion of gauzes or tampons pressuring on the origin of the bleeding. 

Therefore, the development of new pharmacological approaches based on the use of natural or even synthetic agents which actively could block the bleeding and mainly restore a good condition in the nasal epithelium still represents a challenge. Several efforts were made in order to create new pharmacological devices which could match a favorable compliance and encouraging effects in the treatment of epistaxis. In this scenario, this study aims to characterize a mixture of substances which are well-known in the medical field to act separately in different biological manners, as sodium hyaluronate, silver salt, α-tocopherol and D-panthenol. The combination of these four substances was named Emoxilane^®^. 

About the found effects of this composition, we have first assessed the antioxidant activity. Indeed, it is known that the redox signals regulate the fate of cells in health and disease [20]. In this case, the finding of the strong antioxidant effect induced by tocopherol, and here proved on human endothelial and nasal epithelial cells, has represented the first encouraging pharmacological objective. Thus, it was possible to assign an appealing protective action to this composition, already in a preliminary phase. More interestingly, the antioxidant effect found for the tocopherol is related also to the D-panthenol. This pro-vitamin covers an important action guaranteeing the maintenance of a good hydration status of skin and hair if inserted in systemic and topical formulations. Its effects include fibroblast proliferation and accelerated re-epithelialization in wound healing beside protectant, moisturizer, and anti-inflammatory properties [21]. Therefore, several pieces of evidence support the efficacy of D-panthenol when administered topically in a variety of dermatological disorders, including skin abrasions, petty injuries, chronic ulcers, decubital ulcers, anal fissures, skin transplantation, non-severe burns, diaper dermatitis, epithelial lesions, and the prevention and treatment of breast fissures or skin irritations [22]. Very similar activities were revealed for the sodium hyaluronate to which a notable inhibitory role on antithrombin in vitro was also associated. This attractive outcome was described to include extensive fibrin deposition, extreme angiogenesis, and fibroblast-like cell proliferation with an overall increase of the coagulation activity [23]. Furthermore, hyaluronic acid-based hydrogels have exhibited significantly improved hemostatic capability in vivo with normal and hemophilic injuries compared with a commercially available fibrin-based hemostatic agent and prevented abnormal tissue adhesion after hemostasis [24]. Additionally, sodium hyaluronate is able to interact with fibrin clot providing the driving force to organize a three-dimensional matrix and further playing a major role in the subsequent tissue reconstruction processes. This is consistent with the proposed interaction between hyaluronate and fibrin which could function as activator for the clotting reaction and with the induction of proliferation/adhesion of endothelial cells when in presence of formulations based on hyaluronate and synthetic polymers [25,26]. 

Moreover, an enlarged therapy approach for epistaxis also requires the use of intranasal bactericidal creams which often represent a further supplied formulation. This cue becomes important considering that an increased bacterial carriage rate could be a contributory factor in this process [27]. For that reason, one of the substances inserted in Emoxilane^®^ was the silver salt which is notably known, as also proved in this study, to retain a significant bacterial growth-inhibiting power. In particular, the combination of α-tocopherol, D-panthenol and sodium hyaluronate in no way interferes with the activity of the silver salt enhancing how the topical use of the whole association could also act against the bacterial infections. 

## 5. Conclusions

Taken together, these data allowed obtaining fascinating preliminary information about Emoxilane^®^ as a composition of tocopherol, panthenol, sodium hyaluronate, and silver salt for the treatment of epistaxis. Interestingly, each component included in this combination has shown to carry specific effects aiming the antioxidant and antimicrobial functions beside the anti-fibrinolytic and pro-coagulant purposes. Thus, the in vitro analysis we have performed represents an attractive starting point to elaborate further evaluation, mainly in vivo ones, to validate Emoxilane^®^ as an active principle of a new topical formulation for nasal use able to block bleeding. The motivating challenge we have proved to answer addresses the creation of a pharmacological advance which could act on this process, also allowing a good maintenance of nasal endothelium and epithelium, as well as temporary induction of clotting.

## Data Availability

The data presented in this study are available upon request from the corresponding author.
